# Developing Emergency Triage Systems in Cambodia

**DOI:** 10.7759/cureus.11233

**Published:** 2020-10-29

**Authors:** Ayesha Khan, Brian Rice, Peter Acker

**Affiliations:** 1 Department of Emergency Medicine, Stanford University School of Medicine, Palo Alto, USA

**Keywords:** emergency medicine, triage, low resource setting, global health, cambodia, maternal health, child health, development, emergency department

## Abstract

As Cambodia works to rebuild its public health system, an area of focus has been improving the quality of emergency services. After a needs assessment in 2011, project partners identified the implementation of a patient triage system as the first target for development efforts. A context-specific triage system was created using the input of a spectrum of local stakeholders. It was tailored to fit the needs and resources available within the Cambodian health system. The system was implemented through a series of educational interventions at 35 public hospitals throughout nine Cambodian provinces. Follow-up quality improvement visits occurred on a quarterly basis between February 2016 and September 2018, during which feedback on the system was gathered using both quantitative and qualitative methods, and additional system updates were implemented. In this technical report we aim to describe the triage system design, implementation and quality improvement processes utilized with the hope of informing and supporting colleagues working to address similar challenges in other areas of the world.

Through this assessment process a number of key observations were made: 1) Establishment of context-specific emergency triage systems is feasible in low resource settings; 2) Development of new triage processes requires an iterative approach; 3) Successful uptake of new practice systems requires flexibility from both the implementers and end-users in the development relationship; 4) Process improvement requires consistent retraining and reinforcement.

## Introduction

Developing acute care services is identified by the World Health Organization (WHO) as an urgent priority for low- and middle-income countries (LMICs) [1]. Though significant effort was put into restoring Cambodia’s health system after its dismantling by the Khmer Rouge, acute care systems have lagged. With more than a third of patients seeking care at Cambodian health facilities for treatment of acute conditions [2], the Cambodian Ministry of Health (MOH) has recommended prioritization of efforts to strengthen emergency care in the Strategic Health Plan 2008-2015 [3].

To support the MOH’s efforts, the University Research Co. LLC (URC) and Stanford Emergency Medicine International (SEMI) partnered as part of the USAID funded Quality Health Services program (QHS) to understand the factors impeding a given facility’s ability to provide timely intervention to patients with emergent conditions. A series of facility visits, practice observations, and stakeholder interviews were conducted. Relevant field observations included the following: a) patients arriving at health facilities self-determine care-venue (often inappropriately) and require redirection b) patients are evaluated on a first-come-first-served basis and c) clerical tasks, including patient registration, are performed before evaluation by a care provider. Triage systems have been shown to improve emergency care delivery in a variety of resource settings [4,5], however patient triage was not occurring at any of the Cambodian health facilities assessed. Based on these observations, the creation and implementation of a tailored, locally appropriate triage system was identified as a priority to improve care for those suffering from time-sensitive conditions. 

## Technical report

Phase 1: system creation and piloting

Insights into current health system challenges were brought to the MOH and their input on and initial approval for plans to design and implement a triage system to improve identification and timely treatment of ill patients were sought. Though the MOH had been supportive of implementing triage systems, up to this point that had been unable to dedicate the required human and financial resources. The project was able to provide these items, reducing a number of pre-existing barriers, and thus the MOH committed to supporting the project and developing support within the Provincial Health Departments and health facilities.

**Figure 1:**
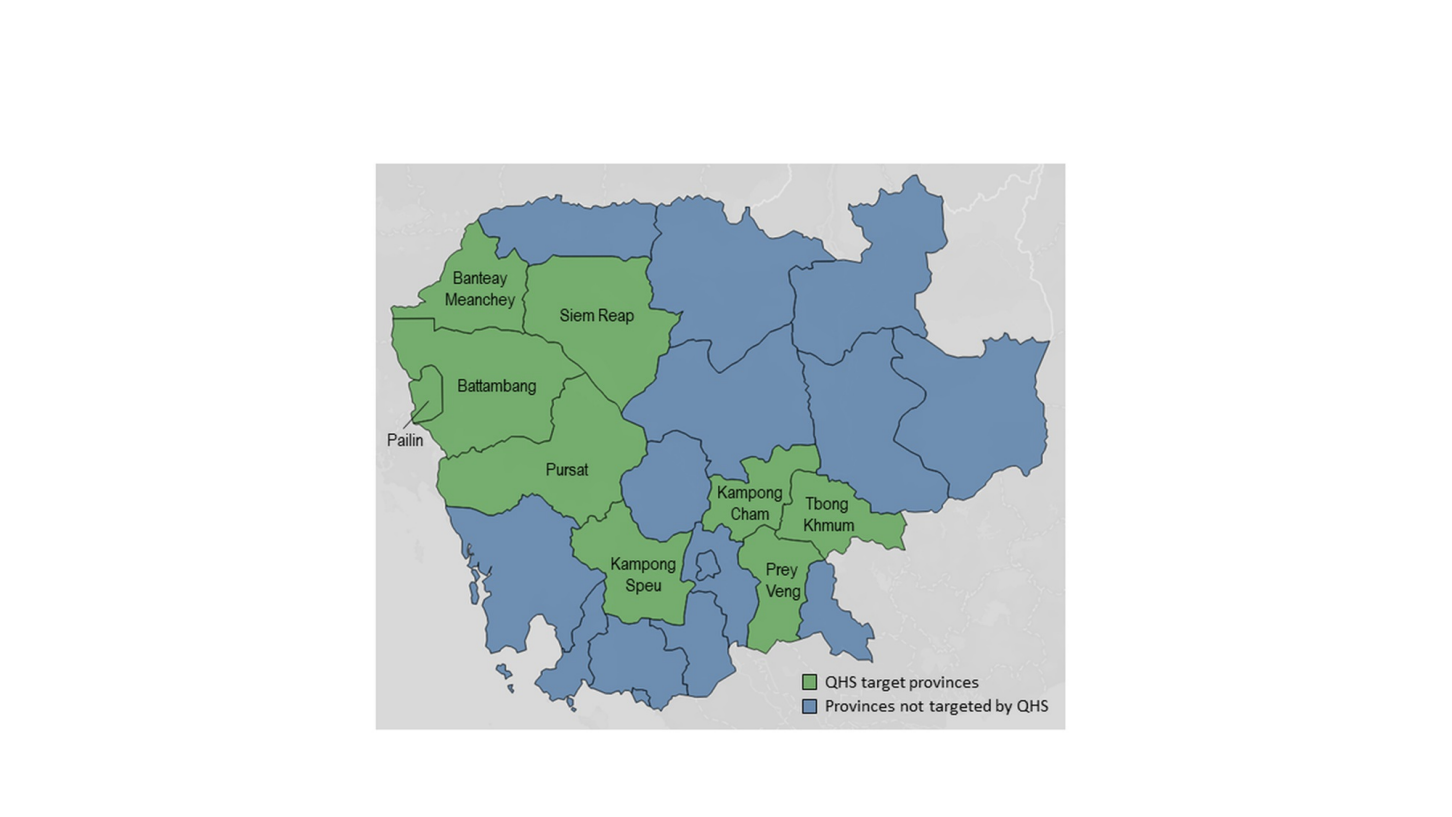
Map of project provinces

After obtaining support from the MOH, triage system design process began in 2012 with project members surveying facility staff and observing practices at five referral hospitals across three provinces: Siem Reap, Battambang, Pursat (see Figure 1 for a map of project provinces). With an understanding of the local needs and, team members evaluated the applicability of the two triage scales developed for low resource settings available at the time, the WHO-developed Emergency Triage Assessment and Treatment (ETAT) [6] program and the South African Triage Scale (SATS) [7]. Both systems provided critical insights, however limitations prevented either system from being applied directly. ETAT is designed to be applied only to pediatric patients, and thus the concepts would need to be expanded to include adults for use in the Cambodian system. The SATS was a relatively new triage tool when OTT was created; it is comprehensive for adults and pediatrics and has been applied with success by a variety of provider types [8]. The application of the SATS does require triaging providers to obtain a full set of vital signs on each incoming patient. After extensive field observations and stakeholder discussions, it was clear that this requirement would create significant challenges in terms of staff time, equipment and training that the triage system implemented would have to take into account. Utilizing elements from both the ETAT and SATS, combined with an understanding of the local health resources and practice environment, a tailored triage system was created.

**Figure 2:**
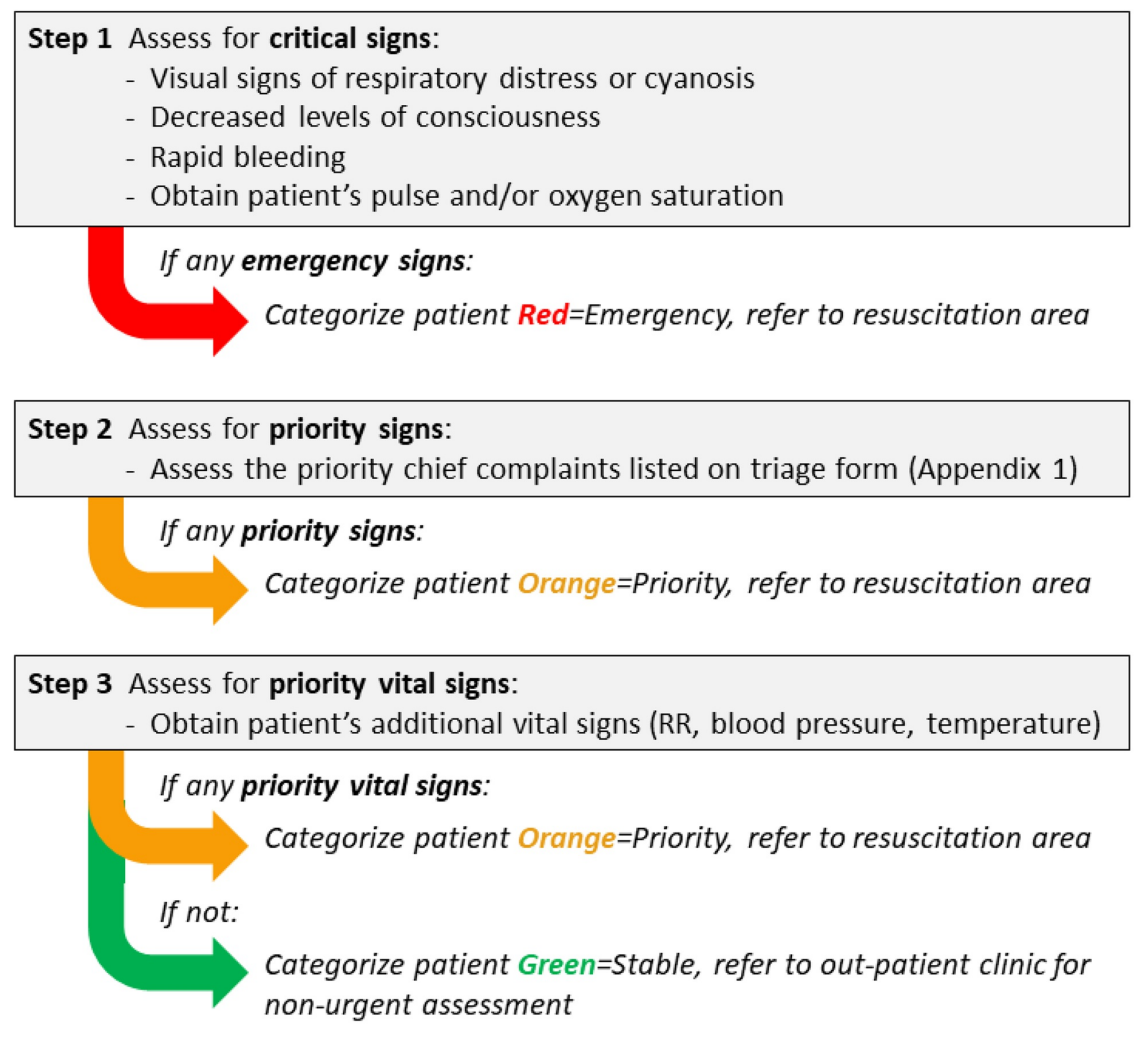
Triage process overview

Guided by a standardized triage form (see Appendices) which would be utilized across all implementation sites and guidelines to identify high-risk vital signs (see Appendices), the system was to be applied by a clinical staff member stationed outside of the emergency care area to all patients arriving at a health facility for an unscheduled visit, including adult, pediatric and maternity patients. The first stage of the triage process separates critical and non-critical patients, with critical patients being immediately shifted to the emergency care area. In most Cambodian health facilities, the emergency care area is staffed by nurses, who would provide the initial assessment and interventions to an incoming patient, and overseen by a doctor who, if not immediately available, could be called to assess an ill patient from another part of the hospital. The remaining non-critical patients enter the second stage of the triage process and are differentiated into urgent and non-urgent categories (see Figure 2 for an overview of the triage process), with urgent patients being directed to the emergency care area, to be seen after patients identified as emergent, and non-urgent patients being sent to the outpatient clinic. Patients referred to the outpatient clinic were seen in the order they arrived, by a clinical staff member, either a physician or a nurse.

The system was tested for reliability and validity, then was piloted in five hospitals using a two-day training curriculum [9]. Day one introduced new tools, theory, and job aids. Day two provided an opportunity for staff to redesign the patient intake process and patient flow to facilitate rapid triage. Extensive feedback was solicited from triage stakeholders at each individual facility on both days of the training through guided discussion, led by project staff. On day one, input was sought related to the overall triage process and how it could be tailored to best suit the needs of a given facility while working within the local resource set. On day two, facility staff and project staff discussed the facility's physical layout, staffing and current workflows to identify means through which to promote the role of effective triage. At the end of each day, project staff met with facility leadership to discuss insights and proposed updates, sharing the forecasted costs and benefits, and working to find solutions acceptable to both parties. These insights were iteratively incorporated into the triage plans for each individual facility, helping to refine the system and adapting it to the needs of each facility, its staff and patients. The perspective gained at each facility was then used to update overall project practices and helped craft the approach to implementation at subsequent facilities. The major challenges encountered during Phase 1 (and subsequent Phases), their root causes, and the solutions to those problems are listed in tabular form in Table 1.

**Table 1:** Challenges, root causes and solutions

Phase 1: System Creation and Piloting (2011 - 2013)		
Challenge	Root Cause(s)	Solution(s)
Lack of stakeholder buy-in	Task saturation of central and provincial health system leadership	Provided outside resources to design and implement locally tailored triage system, minimizing burden on local health system resources
Overcrowding at peak times	Staffing inadequate to efficiently perform complete nursing assessments causing patient backup	Initial triage assessment supplemented by complete nursing assessment performed on wards
Triage staff obtained incomplete vitals	Unclear staff responsibility for taking vitals	Visual cues for vital signs added; final responsibility for vitals placed on triage
Overly complex triage system	Triage had two stages, with the second stage separating urgent and non-urgent patients	Stage Two eliminated; non-urgent patients seen in outpatient clinic
Phase 2: Training and Scaling (2014 - 2016)		
Challenge	Root Cause(s)	Solution(s)
Crowding at centralized intake	Inadequate physical space; inadequate staff at peak times; high patient load at peak times	Equipment/space repurposed for triage; ward staff trained to assist triage; scheduled patients skipped triage during peak hours
Lack of institutional memory of triage procedures	High staff turnover; new hires assigned to triage	Onboarding material and laminated job aids created
Missing equipment	Departing staff brought equipment to new assignments	Nursing leadership designated to store and oversee care of triage equipment
Phase 3: Evaluation and Reinforcement (2016 - 2018)		
Challenge	Root Cause(s)	Solution(s)
Defining evaluation metrics for triage system	Outcome/mortality data unavailable; disposition data inconsistently recorded; self-reported time data incomplete/unreliable	Mixed methods to combine quantitative chart audits with qualitative structured staff interviews

Phase 2: training and scaling

The revised triage system was implemented across 30 additional hospitals throughout nine Cambodian provinces (see Figure 1). As the geographic scope broadened, three regional teams were created to train the clinical staff (typically nurses) selected to implement triage at each of the facilities in their catchment areas. They introduced triage through the two-day training structure and followed it with a reinforcement visit one month after the initial training. Quarterly quality improvement visits were performed throughout the remainder of the project. 

The quality improvement visits showed that successful function of the triage system required a number of key triage promoting conditions: the presence of a centralized triage location, performance of triage prior to registration, presence of appropriate triage equipment, a limited number of patient entrances to a facility, and presence of signs directing patients to the triage area. Originally a standardized design to promote these practices across all facilities was created. However, it became evident that a flexible and facility-specific tailored plan was needed, due to significant inter-facility variability in staffing, layout, equipment and leadership support. Details on this process are found in Table 1.

Phase 3: evaluation and reinforcement

The ultimate goal of implementing this triage system in Cambodia is to reduce the interval between a patient’s arrival and a critical intervention. However, due to a variety of factors, collecting data to assess the triage system's impact on this measure was not feasible. After examining a number of alternative evaluation metrics, a structured triage audit tool was created to gather quantitative triage performance data at quarterly visits. Structured interviews were performed with project staff to elicit qualitative data to better understand the facility factors underlying the performance noted on follow up visits and through the data generated by the triage audit tool. The structured interviews focused on assessing the presence and levels of hospital leadership support for triage, adequate staffing, staff support for triage, and presence of necessary space and equipment to perform triage amongst other related topics. These process indicators could be extrapolated to provide perspective on the system’s impact on patient care. 

The triage audit tool was used to audit 10 random patient charts at each quarterly facility visit, assessing the quality and completeness of the triage form in a standardized, reproducible fashion (see Appendices for an example of the Triage Audit Tool). Triage was considered “complete” if a triage acuity color was assigned to the patient, and “correct” if the triage acuity color assigned was congruent with patient data, as determined by the trained chart auditor. In total, 325 individual quarterly follow up visits, producing 3,250 chart audits, were made to the 35 intervention facilities between February 2016 and September 2018. The first visit was used to establish the performance baseline for comparison in subsequent analysis. Analysis of the data generated by the triage audit tools during these follow-up visits included univariate logistic regression for triage “completeness” and “correctness” and chi-squared analysis of interview responses.

**Figure 3:**
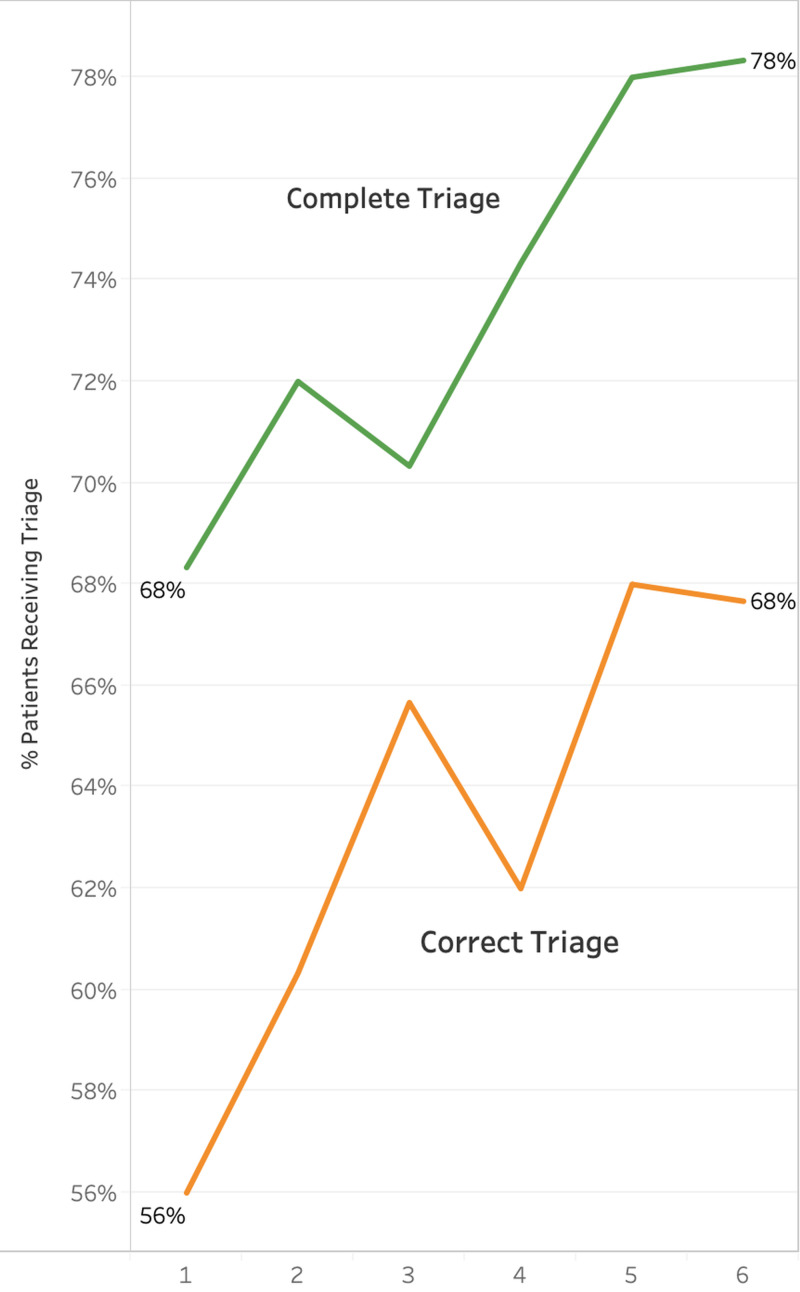
Average triage performance amongst all facilities per quarter

Amongst all facilities, the percentage of patients receiving “complete” triage and “correct” triage improved significantly both statistically and clinically from the beginning to the end of the follow-up period. These results are displayed graphically in Figure 3.

The results of the structured interviews were analyzed to identify themes that correlated with either strong or poor triage performance. Each facility had their triage performance assessed individually, using the triage audit tool data, and were ranked into three tiers based on their triage performance (highest, average, and lowest) based on how frequently they produced “complete” and “correct” triage. When the survey results for each of these performance groups were assessed, a number of correlations were noted. All eight of the lowest-performing tier facilities reported a lack of hospital leadership support for triage. Staff was described as supportive in seven out of the nine highest performing tier facilities, but in only one out of the eight lowest-performing tier facilities. Only two facilities reported a lack of basic equipment and they were both in the lowest tier.

## Discussion

Operationalizing triage in resource-limited settings is a critical step in strengthening acute care systems and improving patient outcomes [10]. However, changing practice patterns to accommodate triage in resource-limited settings is complex, because it necessitates a cultural change that prioritizes obtaining clinical information over clerical information during the intake process. Particularly in Cambodia, a cultural change that enforced medically appropriate venues for care over patient self-direction was also necessary, as was standardizing a system of hospitals that is variable in staffing models and infrastructure. Our project demonstrated that standardized triage can be implemented and maintained in Cambodian government hospitals despite these challenges. Our analysis of these efforts suggested that the project was successful in continuously improving the quality of triage being performed (see Figure 3).

Four key factors to success were identified during quality improvement field visits. First, participatory development of the triage system during the pilot and iterative changes during implementation created feelings of ownership for Cambodian partners and allowed for a context-specific system to emerge. Second, buy-in from health system and hospital leadership was absolutely essential, as these stakeholders held the decision making power that granted access to all the other resources required to set up the physical elements and the workflows required to implement a triage system. Third, workforce capacity was optimized allowing for dedicate clinical staff (typically nurses) to be posted at a single facility entrance during busy times and flexible staffing allowing ward nurses to triage patients on the wards during low volume hours. Finally, repeated reinforcement of both the importance of triage as well as the skills needed for using the triage system during follow-up visits was vital to success. 

Our analysis of survey results highlighted consistent/supportive triage staff, presence of necessary equipment and, echoing the findings above, hospital leadership support as elements that promoted successful triage. Primarily, hospital leadership facilitated the culture change necessary to support implementation. Likely, the strong hierarchical decision-making structure encountered in Cambodia allows staff to feel comfortable enforcing their medical decision-making when they had the assurance that their facility leadership stood behind the triage system. Staff ownership also comes with management support. Similarly, staff enthusiasm for intake and flow changes correlated with the success of implementation; where providers were hesitant to learn a new system, triage success lagged. Lastly, having access to the equipment required to perform the process of triage (a designated area with vital sign taking equipment and triage forms) was essential to effective triage implementation. 

As efforts proceed in Cambodia and similarly resourced settings to support the development of emergency care systems, including implementation of triage systems will be essential in improving access to quality emergency care. The WHO has invested significant resources in this area, and has recognized the importance of triage particularly. They, along with the International Committee of the Red Cross and Medecins Sans Frontieres, have collaboratively developed a triage system designed for resource-limited settings. This triage systems is being rolled out as part of their package of emergency care system strengthening tools, which will aid in the dissemination of these essential elements health system components to areas in which they are currently lacking [11].

## Conclusions

Our experience demonstrated that establishment of emergency triage systems is feasible in low resource settings, like Cambodia. The iterative approach utilized allowed for real-world constraints to be identified and incorporated into triage practice, and local health system intricacies to be adapted to. This approach was essential to ensuring the triage system fit the needs of the end users and the realities of the Cambodian health system it was intended to support.

To promote the systems ongoing use, as the project neared completion efforts were made to transition the duties of triage support to MOH and Provincial Hospital Directors’ (PHDs) staff, providing them with additional training and a comprehensive package of triage education and evaluation materials. 

The MOH and PHDs have both demonstrated a commitment to continue support for triage systems in Cambodia. In addition to providing technical expertise and political support, the MOH has incorporated elements of triage into national guidelines governing hospital functions while many PHDs provided ongoing staffing support for triage follow up visits. With the QHS project now having concluded, the system’s ongoing use will depend on the MOH and PHDs ability to support it, and project partners remain optimistic.

## Figures and Tables

**Figure 4:**
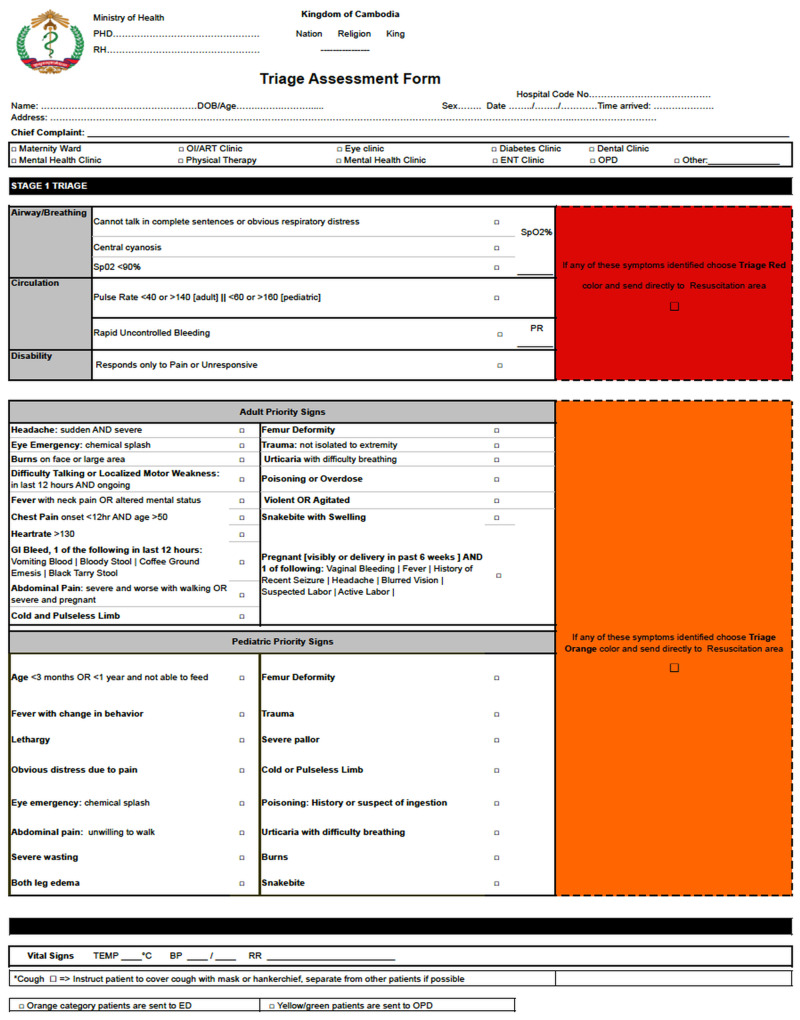
Standardized triage form (English version)

**Figure 5:**
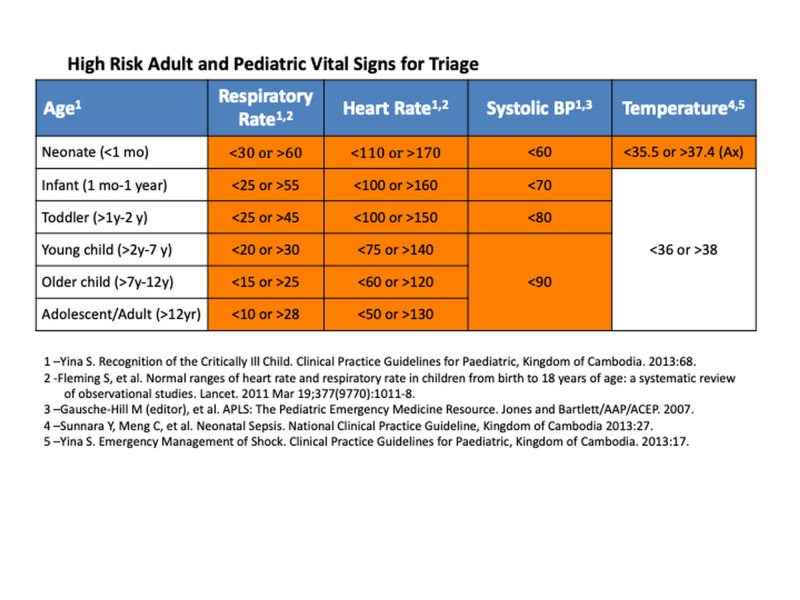
High risk adult and pediatric vital signs for triage

**Figure 6:**
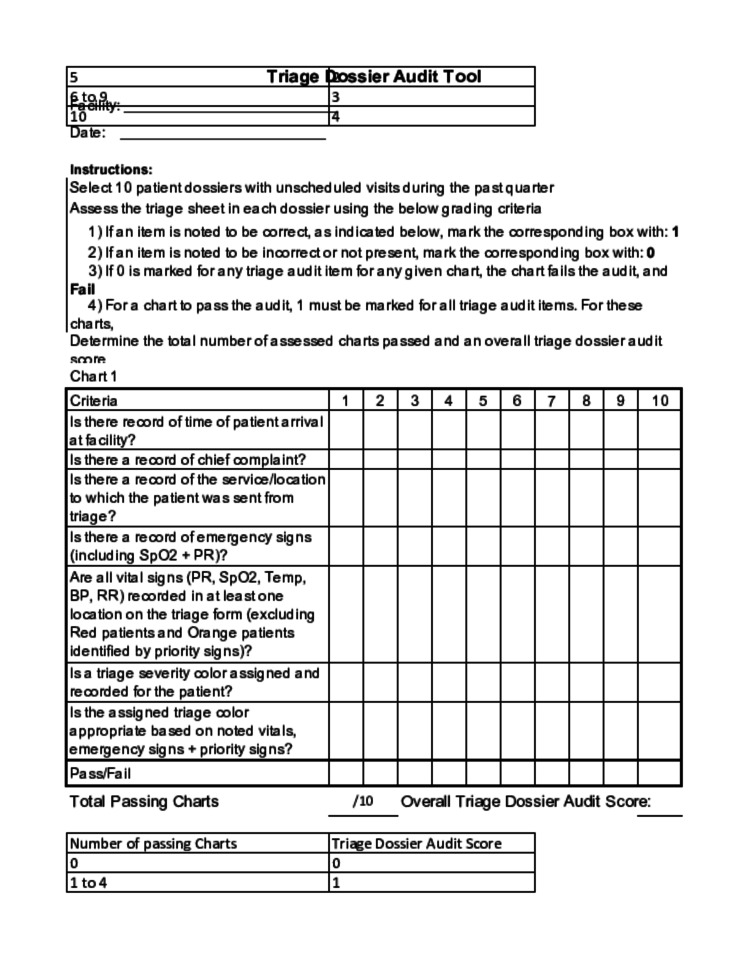
Triage audit tool
